# Environmental and occupational respiratory diseases – 1041. The evaluation of the role of obesity on adults with bronchial asthma

**DOI:** 10.1186/1939-4551-6-S1-P40

**Published:** 2013-04-23

**Authors:** Tanja Grzetic-Romcevic, Silvana Sonc, Boris Devcic, Tanja Grzetic-Romcevic

**Affiliations:** 1 Pulmonary, Hospital Sezana, Sezana, Slovenia; 2 Clinical Department for Pulmonary Diseases and Allergy, University Clinical Center, Ljubljana, Ljubljana, Slovenia

## Background

Data on the association between obesity and asthma outcomes are clinically important and not always well understood. AIM: to evaluate the impact of obesity on asthma control and clinical course of the disease.

## Methods

Between January 2011 and December 2011, a total of 293 consecutive asthmatic adult patients (56.7% female, 43.3% male) attending to our out-patients clinic (Sezana) were involved in the study. The diagnosis of asthma was assessed according to Global Initiative for Asthma (GINA) guidelines. Asthma control was assessed by Asthma Control Test (ACT). Information about obesity was collected by measuring BMI (Body Mass Index). Obese patients were defined as a patients with the body mass index >30.

## Results

The mean age value was 47+- 4.1years. 76 patients (26.1%) had BMI<25kg/m2, 87 patients (29.9%) had BMI 25- 29 kg/m2 and 130 patients (44%) had BMI >30 kg/m2. Almost half of our investigated patients with bronchial asthma were obese. Obese patients were more often female (60% vs 40%). According to Asthma Control test scores 67.7% of our patients had controlled and 32.3% had uncontrolled asthma (15.8% partly controlled and 16.5% uncontrolled). The reverse situation were among obese patients. There were more obese patients in the not well controlled group (33.1% had uncontrolled and 27.9% had partly controlled asthma) than in the controlled ones (39%). The number of asthmatic patients requiring hospitalization or emergency room visit due to asthma (in the investigated period) was higher in obese patients (6.4% and 18.6% respectively), than in nonobese asthmatic patients (1.8% and 1.6% respectively).

## Conclusions

Our results suggest that obesity is associated with more severe and less controlled asthma, with poor clinical courses.

